# Physiological effects of five different marine natural organic matters (NOMs) and three different metals (Cu, Pb, Zn) on early life stages of the blue mussel (*Mytilus galloprovincialis*)

**DOI:** 10.7717/peerj.3141

**Published:** 2017-04-12

**Authors:** Lygia Sega Nogueira, Adalto Bianchini, Scott Smith, Marianna Basso Jorge, Rachael L. Diamond, Chris M. Wood

**Affiliations:** 1 Department of Zoology, University of British Columbia, Vancouver, British Columbia, Canada; 2 Department of Biology, McMaster University, Hamilton, Ontario, Canada; 3 Bamfield Marine Sciences Centre, Bamfield, British Columbia, Canada; 4 Instituto de Ciências Biológicas, Universidade Federal do Rio Grande—FURG, Rio Grande, Rio Grande do Sul, Brazil; 5 Department of Chemistry and Biochemistry, Wilfrid Laurier University, Waterloo, Ontario, Canada

**Keywords:** Doc, Ca^2+^+Mg^2+^-ATPase, Lipid peroxidation, Carbonic anhydrase

## Abstract

Metals are present in aquatic environments as a result of natural and anthropogenic inputs, and may induce toxicity to organisms. One of the main factors that influence this toxicity in fresh water is natural organic matter (NOM) but all NOMs are not the same in this regard. In sea water, possible protection by marine NOMs is not well understood. Thus, our study isolated marine NOMs by solid-phase extraction from five different sites and characterized them by excitation-emission fluorescence analysis—one inshore (terrigenous origin), two offshore (autochthonous origin), and two intermediate in composition (indicative of a mixed origin). The physiological effects of these five NOMS alone (at 8 mg/L), of three metals alone (copper, lead and zinc at 6 µg Cu/L, 20 µg Pb/L, and 25 µg Zn/L respectively), and of each metal in combination with each NOM, were evaluated in 48-h exposures of mussel larvae. Endpoints were whole body Ca^2+^+Mg^2+^-ATPase activity, carbonic anhydrase activity and lipid peroxidation. By themselves, NOMs increased lipid peroxidation, Ca^2+^+Mg^2+^-ATPase, and/or carbonic anhydrase activities (significant in seven of 15 NOM-endpoint combinations), whereas metals by themselves did not affect the first two endpoints, but Cu and Pb increased carbonic anhydrase activities. In combination, the effects of NOMs predominated, with the metal exerting no additional effect in 33 out of 45 combinations. While NOM effects varied amongst different isolates, there was no clear pattern with respect to optical or chemical properties. When NOMs were treated as a single source by data averaging, NOM had no effect on Ca^2+^+Mg^2+^-ATPase activity but markedly stimulated carbonic anhydrase activity and lipid peroxidation, and there were no additional effects of any metal. Our results indicate that marine NOMs may have direct effects on this model marine organism, as well as protective effects against metal toxicity, and the quality of marine NOMs may be an important factor in these actions.

## Introduction

Metals are naturally present in the aquatic environment; however, depending on their concentration, availability and residence time they have the potential to cause animal toxicity, especially in pollution-impacted environments (Wood, 2012). In freshwater, acute metal toxicity is related to the competition for ionic binding sites in the epithelial membranes and enzymatic inhibition mainly at the gill cells of aquatic animals (Di Toro et al., 2001; Niyogi & Wood, 2004; Dudev & Lim, 2014). Similar mechanisms are thought to occur in seawater organisms, though there have been only a few mechanistic studies (Lopes et al., 2011; Martins et al., 2011; Nogueira et al., 2013; Tellis et al., 2013; Tellis et al., 2014). Copper (Cu) is considered one of the most toxic metals to aquatic organisms, and based mainly on studies with freshwater fish, it affects sodium (Na^+^) transport sites (apical Na^+^ transporters and basolateral Na^+^, K^+^-ATPase) and therefore Na^+^ homeostasis (reviewed by Grosell, 2012). Zinc (Zn, reviewed by Hogstrand, 2012) and lead (Pb, reviewed by Mager, 2012) also cause ionoregulatory dysfunction but mainly for calcium (Ca^2+^) homeostasis because calcium transport sites (apical Ca^2+^ channels and basolateral Ca^2+^-ATPase) are impacted by these metals. Nevertheless, these may not be the only mechanisms of toxicity, and there is emerging evidence that metals may also induce oxidative stress (Lushchak, 2011) and inhibit a key gill enzyme, carbonic anhydrase (Wood, 2012; Zimmer et al., 2012).

It is now clear that one of the most important factors influencing metal toxicity, at least in fresh water, is natural organic matter (NOM, usually measured as dissolved organic carbon, DOC in mg C/L), and that all NOMs are not the same in this regard (Al-Reasi, Wood & Smith, 2011; Wood, Al-Reasi & Smith, 2011). Traditionally, the protection against metal toxicity to aquatic animals has been attributed to the ability of NOMs to bind metals, thereby reducing their bioavailability (Thurman, 1985; McGeer et al., 2002; Nadella et al., 2009). The optically dark, more aromatic NOMs of “allochthonous” origin (also known as “terrigenous”—i.e., from land) appear to be more effective in this regard than the paler, less aromatic NOMs of “autochthonous” origin (i.e., generated by algal and microbial metabolism within the water column) (Perdue, 1998; Xue & Sigg, 1999; Kogut & Voelker, 2001; Tipping, 2002; Schwartz, Curtis & Playle, 2004; De Schamphlelaere & Janssen, 2004; Baken et al., 2011; Al-Reasi, Wood & Smith, 2011). However there is also evidence, summarized by Wood, Al-Reasi & Smith (2011), that NOMs in themselves may exert positive physiological effects on freshwater organisms, thereby contributing to protection, and that again the darker terrigenous NOMs are the most potent (e.g., Galvez et al., 2008; Duarte et al., 2016).

In sea water, there is now evidence that NOM strongly protects against Cu toxicity, but the particular source or chemical nature of the NOM may or may not have a large influence (Arnold, 2005; Arnold, Santore & Cotsifas, 2005; Arnold, Cotsifas & Corneillie, 2006; Rosen et al., 2008; DePalma et al., 2011a; DePalma et al., 2011b; Cooper et al., 2014). On the other hand, two NOMs (one of terrigenous freshwater origin, the other isolated from inshore sea water) were only weakly protective against Pb toxicity in sea water, and did not alter Zn toxicity (Nadella et al., 2013). These conclusions were based on early life stage development tests with the larvae of blue mussels, which are among the most sensitive organisms to metal toxicity in the marine environment (US EPA, 2003; Arnold et al., 2010). However, in another very sensitive organism, the developing larvae of the purple sea urchin, these two NOMs actually caused toxicity by themselves (Nadella et al., 2013), a phenomenon which has also been seen in mussel larvae exposed to high concentrations of terrigenous freshwater NOMs (Nadella et al., 2009). However, the relevance of the effects of freshwater NOMs in the marine environment is debatable.

The objectives of the present study were to test the influence of a range of true seawater NOMs actually collected from the marine environment on physiological indices of toxicity, in the presence or absence of sublethal concentrations of Cu, Pb, or Zn, using developing larvae of the blue mussel, *Mytilus galloprovincialis*. These organisms are cultured commercially on the west coast of Vancouver Island, British Columbia, close to the sites where most of the NOMs were collected. The various NOMs were characterized by excitation-emission fluorescence (Nadella et al., 2009; Gheorghiu et al., 2010; DePalma et al., 2011a; DePalma et al., 2011b; Tait et al., 2016). The sites selected for collection ranged from inshore to offshore, and according to fluorescence analysis, the resulting NOM isolates ranged from terrigenous to autochthonous. The endpoints selected were carbonic anhydrase activity, Ca^2+^+Mg^2+^-ATPase activity, and lipid peroxidation, the latter as an indicator of oxidative stress. Our specific hypotheses were: (i) that at least some of the NOMs would exert positive effects on the organism; (ii) that metals alone would exert negative effects, which would differ according to the metal; (iii) that at least some of the NOMs would be protective, ameliorating the negative effects of the metals; and (iv) that these effects would vary both amongst the different NOMs, and among the different metals.

## Material and Methods

### Extraction and characterization of NOMs

NOM extraction followed the solid-phase method described by Rodriguez & Bianchini (2007). Approximately 200 L of water were collected from five different field sites and transferred to the laboratory. The samples were named: Bamfield (48°48′54.0″N; 125°09′32.1″W), Port (48°49′47.8″N; 125°07′36.1″W), Pachena (48°50′17.4″N; 25°08′12.3″W), Offshore Canada (48°50′41.4″N; 125°08′37.2″W) and Offshore Brazil (32°18′16.0″S; 51°45′28.3″W). The water from each field site was sequentially filtered using 1.0 and 0.5 µm mesh filters (Cuno, Polyclean^®^, Brazil) and acidified to pH 2 with HCl. The acidified water was passed through PPL cartridges (Mega Bond Elut PPL, 5 GM 60 mL, 16/PK; Varian, Georgia, CAN) to remove highly polar species. The salts retained in the PPL cartridges were removed using acidified ultrapure water (pH 2). At the end of the process, the NOM elution was performed using methanol that was then removed by lyophilization, and the concentrated NOM solutions were prepared with ultrapure water. Activation of the PPL cartridges, removal of salts, and rinsing, as well as NOM elution, lyophilization, and storage were performed as described by Rodriguez & Bianchini (2007) and Dittmar et al. (2008). A small volume of each of the NOMs was filtered using 0.45 µm mesh filters (Steritop^®^ filter; Millipore, Billerica, MA, USA), diluted appropriately, and the dissolved organic carbon (DOC) was measured (TOC-V CSH, Shimadzu, Kyoto, Japan).Test solutions were similarly assayed for DOC, without dilution. The five NOMs from different sources were stored in the dark at 4 °C.

Fluorescence spectra were measured on the five different NOM sources as indices of quality. Fluorescence spectra of the NOM samples were collected using a fluorescence spectrophotometer (Varian Cary, Palo Alto, CA, USA) with 1 cm path-length quartz cuvettes. The fluorescence spectra were measured at excitation wavelengths from 200 to 450 nm using 10 nm increments, and emission wavelengths in the range of 250–650 nm in 1 nm increments. Each matrix contains fluorescence information for specific excitation/emission wavelength pairs. The excitation-emission matrices EEMs were plotted as contours to identify the fluorescing components. Parallel Factor Analysis (PARAFAC) was then used to identify and quantify the component peaks in the EEMs, as implemented in the PLS Toolbox (Eigen vectors Research Inc, Mason, WA, USA) running on a Matlab™ platform. PARAFAC resolves this information into component spectra and relative component concentrations (Nadella et al., 2009; Gheorghiu et al., 2010; DePalma et al., 2011a; DePalma et al., 2011b). PARAFAC assigned the fluorescence on a percentage basis based on the *a priori* assumption that there were three components (humic substance-like, tyrosine-like, and tryptophan-like; for details, see Al-Reasi, Wood & Smith (2011), Al-Reasi, Smith & Wood (2012), Al-Reasi, Wood & Scott (2013), and Tait et al. (2016). Note that in the cited works often four components were used and humic substances were separated into humic and fulvic acid components. For the analysis here, it was found to be difficult to resolve humic and fulvic acids separately so the long wavelength, non-protein, component is referred to as humic substances which can be viewed as the sum of humic and fulvic acids. As an additional source differentiation measure, the fluorescence index was calculated as the ratio of fluorescence emission measured at 450 nm divided by emission at 500 nm for an excitation of 370 nm (McKnight et al., 2001).

### Collection of gametes and fertilization

Adults of the mussel *M. galloprovincialis* were obtained by courtesy of Northwest Aquaculture Farm located on Effingham Inlet in Barkley Sound on the west coast of Vancouver Island. At Bamfield Marine Sciences Centre (BMSC), they were held in 10 L tanks at 10–15 °C in aerated seawater (30 ppt). Males and females were induced to spawn through the heat shock method, according to American Society for Testing and Materials (ASTM) (2004). The adult animals were maintained in groups at 22–25 °C until individuals started to release gametes. At this point, the animals were separated into individual beakers containing 200 ml of natural sea water and gamete viability checked. Fertilization was initiated when eggs and sperm from several animals were pooled into a single beaker containing 0.2 µm filtered 30-ppt sea water (∼350 mL). Fertilization success was determined through a microscope, identifying the cleavage initiation (2-cell or 4-cell stage). Once 90% of the eggs were fertilized, the density of embryos per liter was determinated by counting 1 ml under the microscope. The beaker was kept aerated for at least 1 h, the time required for total fertilization and to count the embryos.

### Experimental design

Embryos of *M. galloprovincialis* were exposed to isolated NOMs from each of the five different sources or combined treatments of NOMs and metals (Table S1). All treatments were performed in triplicate (i.e.,  *N* = 3 flasks of approximately 2,500 embryos each) starting at about 1 h post-fertilization. For all the tests, the embryos were transferred into seawater exposure solutions (total volume 250 mL) at a density of 10 individuals/mL, 15 °C for 48 h. These parameters were based on the acute toxicity test protocol with embryos of saltwater bivalve molluscs (American Society for Testing and Materials (ASTM), 2004). The isolated NOM exposures were conducted adding Bamfield, Port, Pachena, Offshore-CA and Offshore-BR NOMs at a nominal concentration of 8 mg DOC/L in 250 mL of BMSC sea water. The combined NOM-metal exposures were performed by adding NOM (8 mg DOC/L) and either 6 µg Cu/L or 20 µg Pb/L or 25 µg Zn/L. Table S1 provides a summary of the experimental treatments. These metal concentrations were chosen as estimated EC20 values in BMSC sea water. These were based on measured EC20 values (7 µg Cu/L) in the same sea water for Cu for the congeneric *Mytilus trossolus* (Nadella et al., 2009), measured EC20 values (16–27 µg Pb/L) in the same sea water for Pb for both *M. galloprovincialis* and *M trossolus* (Nadella et al., 2013), but preliminary estimates for Zn. Later measured EC20 values for Zn for the two species in the same sea water were higher (69–101 µg Zn/L; Nadella et al., 2013), so the chosen value for Zn corresponded to only about EC5. In addition to these treatments, embryos of *M. galloprovincialis* were exposed in BMSC sea water without addition of NOMs or metals (control condition), and also exposed to single metals in Bamfield sea water without addition of NOMs. At the end of 48 h exposure, the samples were filtered using an 8-µm polycarbonate filter (Nucleopore Track-Etch Membrane PC MB 47 mm; Whatson PLC, Maidstone, Kent, UK) under gentle vacuum, and the D-veliger larvae were collected in 1.5-mL vials and stored at −80 °C for later analyses.

### Ca^2+^ + Mg^2+^-ATPAse activity

Samples were homogenized in a buffer solution (in mM: 150 sucrose; 50 imidazole; pH: 7.6) for measurement of both Ca^2+^ + Mg^2+^-ATPase and carbonic anhydrase activities (see below). Ca^2+^ + Mg^2+^-ATPase measurements were performed using an assay originally described by Vajreswari et al. (1983). The method is based on the difference between the amounts of inorganic phosphate (Pi) released before and after incubation (30 min) in specific media, both lacking K^+^, but one with Mg^2+^ present but no Ca^2+^, and the other with both Ca^2+^ and Mg^2+^ present. The first is intended to yield Mg^2+^ ATPase activity, and the difference between the two is intended to yield Ca^2+^ATPase activity. In our hands, the two incubation media generally yielded very similar activities such that Ca^2+^ATPase activity could not be reliably distinguished. This suggests that the enzyme in blue mussel larvae is rather unspecific in its substrate specificity, being activated in the presence of either divalent cation. Therefore, we have reported only the results obtained in the medium with both cations present (in mM: 80 NaCl; 5 CaCl_2_; 5 MgCl_2_; 20 Tris Base; 3 ATP; 1 ouabain; pH 7.6) as “Ca^2+^+Mg^2+^-ATPase activity”. Pi concentration was determined using a commercial reagent kit (Fosfato; Doles, Goiânia, GO, Brazil). Protein concentration in the homogenate of larvae was determined using the method of Bradford (1976). The specific enzyme activities were expressed as µmol Pi/mg protein/h.

### Carbonic anhydrase activity

Carbonic anhydrase activity in the larvae of the *M. galloprovincialis* was assayed as described by Henry (1991). The method is based on the catalysis of the hydration of the carbon dioxide present in a CO_2_-saturated solution by the enzyme with subsequent release of H^+^ and consequent reduction of the pH. The larvae were homogenized in the same buffer solution used for Ca^2+^+Mg^2+^-ATPase measurements, with the protein concentration again determined by the method of Bradford (1976). An aliquot was added to the reaction solution (in mM: 225 mannitol; 75 sucrose; 10 Tris Base; 10 Na_2_HPO_4_) and an aliquot of a CO_2_ saturated solution was added. The pH was measured every 5 s up to 30 s. The enzyme activity was calculated based on the slope of the linear regression for the pH change over time and the protein content in the cell homogenate. The specific enzyme activity was expressed as an arbitrary unit of carbonic anhydrase (slope of the linear regression)/mg protein/h.

### Measurement of thiobarbituric acid-reactive substances (TBARS)

The extent of lipid peroxidation was determined by the malondialdehyde (MDA) reaction produced during the lipoperoxidation process with thiobarbituric acid, according to Oakes & Van der Kraak (2003). Briefly, larvae samples were homogenized in 1.15% KCl containing 35 mM butylated hydroxytoluene (BHT). The homogenate of larvae was added to the reaction mixture containing sodium dodecyl sulfate (SDS, 8.1%), 20% acetic acid (pH 3.5), thiobarbituric acid 0.8% (TBA), and 67 mM BHT. The mixture was incubated at 95 °C for 30 min. Then, ultrapure water (100 µL) and n-butanol (500 µL) were added and centrifuged at 3,000 rpm for 10 min at 15 °C. The organic phase was placed in a microplate reader, and the fluorescence registered. Protein concentration in the larval homogenate was determined using the method of Bradford (1976). The concentration of thiobarbituric acid reactive substances (TBARS) was expressed as nmol MDA/mg protein.

### Statistical analyses

Data are expressed as means ± standard error (*n* = 3). Each sample (*n*) represents a pool of ∼2,500 larvae of *M. galloprovincialis.* Isolated effects of NOM exposures (without addition of metals) in larvae were calculated by one-way analysis of variance (ANOVA) followed by Tukey’s test. ANOVA assumptions (data normality and homogeneity of variances) were checked and the data transformed as necessary to satisfy these assumptions. For simplicity, as there are so many possible comparisons, the differences among NOMs have not been marked. Thus, the statistical differences were indicated only for comparisons between specific NOMs and the absolute control condition (Bamfield sea water with no added NOM) using an asterisk (*). The effect of metal addition in each treatment (No NOM and all NOMs tested) was evaluated by Student’s two tailed *t*-tests, comparing the presence and absence of metal in the exposure within the same source of NOM. Significant differences have been marked with # signs. The significance level adopted was 95% (*α* = 0.05).

## Results

### Organic matter characterization

**Figure 1:**
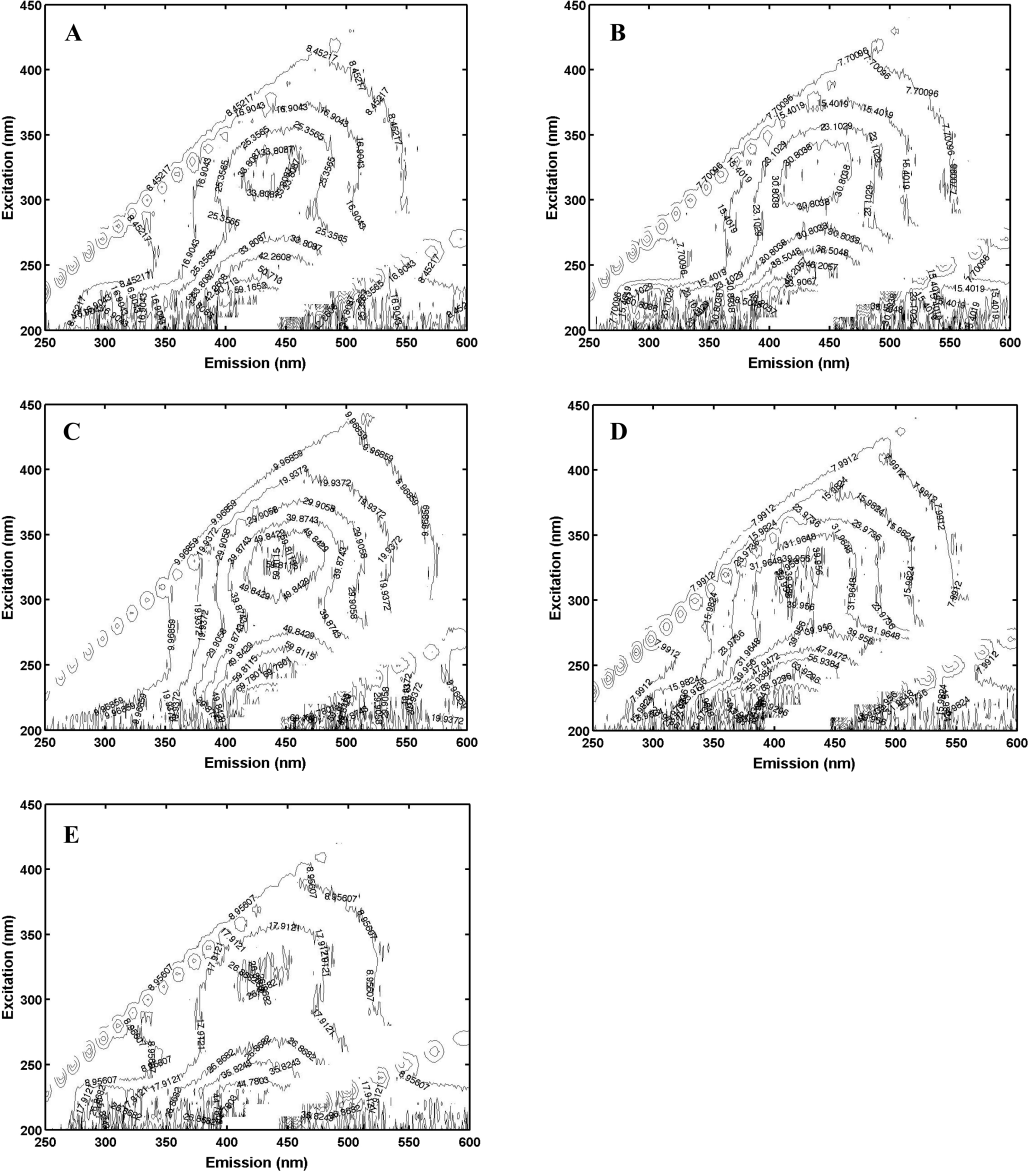
Excitation-emission matrices (“fingerprints”) of each of (A), Port; (B), Bamfield; (C), Pachena; (D), Offshore-CA; (E), Offshore-BR.

Pachena (Fig. 1C) exhibited fluorescence that was based on over 80% humic components with less than 10% each of tryptophan and tyrosine (Table 1); it was therefore classified as the most allochthonous (terrigenous) NOM. Offshore-CA (Fig. 1D) and Offshore-BR (Fig. 1E) both contained less humic-like components and more of the proteinaceous materials (tryptophan-like and tyrosine-like) (Table 1) and were both classified as more autochthonous NOM. Offshore-BR had the greatest relative contribution of tryptophan to the total fluorescence and Offshore-CA had the greatest protein contributions to fluorescence. Finally, Bamfield (Fig. 1B) and Port (Fig. 1A) were classified as more “mixed” terrigenous and autochthonous NOMs with a relatively high amount of tryptophan-like and tyrosine-like components, 20% and 15–20% respectively, with the rest being humic-like material (Table 1). Similar classifications of the sources resulted from looking at the fluorescence index (FI) which was higher for more authochthonous sources and lower for terrigenous sources (Table 1). Both offshore samples (Offshore-CA and Offshore-BR) had the highest values at 1.52 and 1.68 respectively. Port, Bamfield and Pachena had similar values of 1.18, 1.21 and 1.23 respectively.

**Table 1:** Measured dissolved organic carbon (DOC) concentrations, fluorescent index values (FI) and composition values (%) of the NOMs by PARAFAC analysis from different sources.

	DOC (mg/L)	Fluorescent index (FI)	Composition values (%)		
			Trp	Tyr	HS
Port	7.9	1.18	20.6	12.4	55.6
Bamfield	6.3	1.21	20.3	19	60.5
Pachena	7.4	1.23	10	4.5	85.9
Offshore-CA	6.0	1.52	23.7	20.6	55.6
Offshore-BR	9.1	1.68	28.5	10	62.2

**Notes.**

TITLE Trp tryptophan Tyr tyrosine HS humic substances

The measured dissolved organic carbon concentrations in the experimental trials with added NOM ranged between 6–9 mg DOC/L (Table 1). Fig. S1 provides a visual representation of the colours of the different NOMs.

### Isolated effects of NOMs

**Figure 2:**
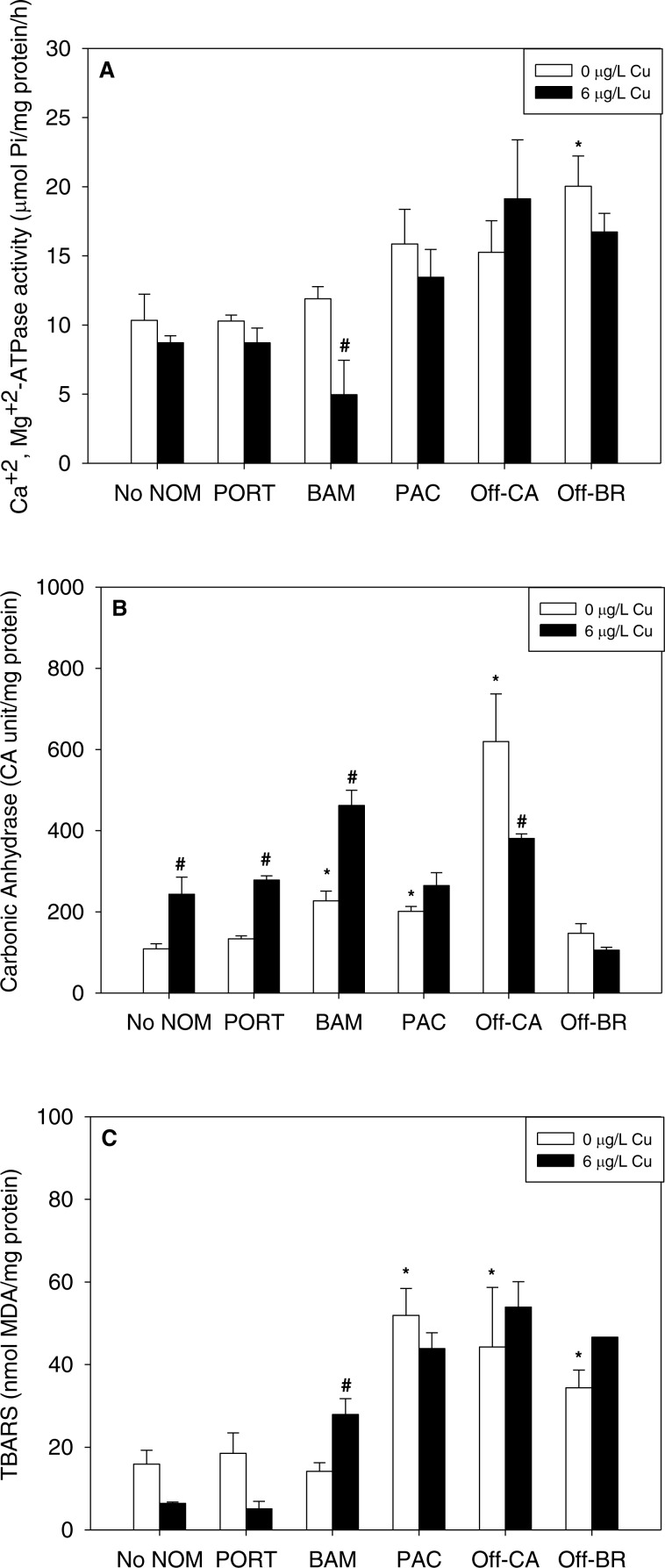
(A) Ca^2+^, Mg^2+^-ATPase activity, (B) carbonic anhydrase activity, and (C) lipid peroxidation, quantified as TBARS, of *M. galloprovincialis* larvae exposed to copper (6 µg/L) for 48 h at the beginning of development. The control condition (Bamfield sea water with no added NOM or metal) and isolated NOM exposures alone (no added metal) are represented by open bars. Treatments in which copper was added are represented by black bars. Mean values which are significantly different from their respective NOM alone controls are marked with #; mean values for isolated NOM exposures alone which are significantly different from the absolute control condition are represented by an asterisk. PORT, Port; BAM, Bamfield; PAC, Pachena; Off-CA, Offshore Canada; Off-BR, Offshore Brazil. Data are means ± standard error (*N* = 3 replicates of 2,500 larvae each).

NOM exposure by itself induced substantial physiological effects in mussel early life stages, significant in seven of 15 NOM-endpoint combinations (marked with * in Fig. 2), effects that were dependent on the source of the organic matter. In comparison to sea water lacking added NOM, autochthonous NOM (Offshore-BR and Offshore-CA) significantly increased lipid peroxidation (Fig. 2C) and the activities of either Ca^2+^+Mg^2+^-ATPase (Offshore-BR; Fig. 2A) or carbonic anhydrase (Offshore-CA; Fig. 2B). Carbonic anhydrase activity and LPO damage were also increased by terrigenous NOM (Pachena). Finally, the two “mixed” NOMs (Bamfield and Port) exhibited no actions by themselves, except for a significant increase in carbonic anhydrase activity caused by Bamfield (Fig. 2B). *P*-values with statistical significance have been appended in the (Table S2).

### Isolated effects of metals

**Figure 3:**
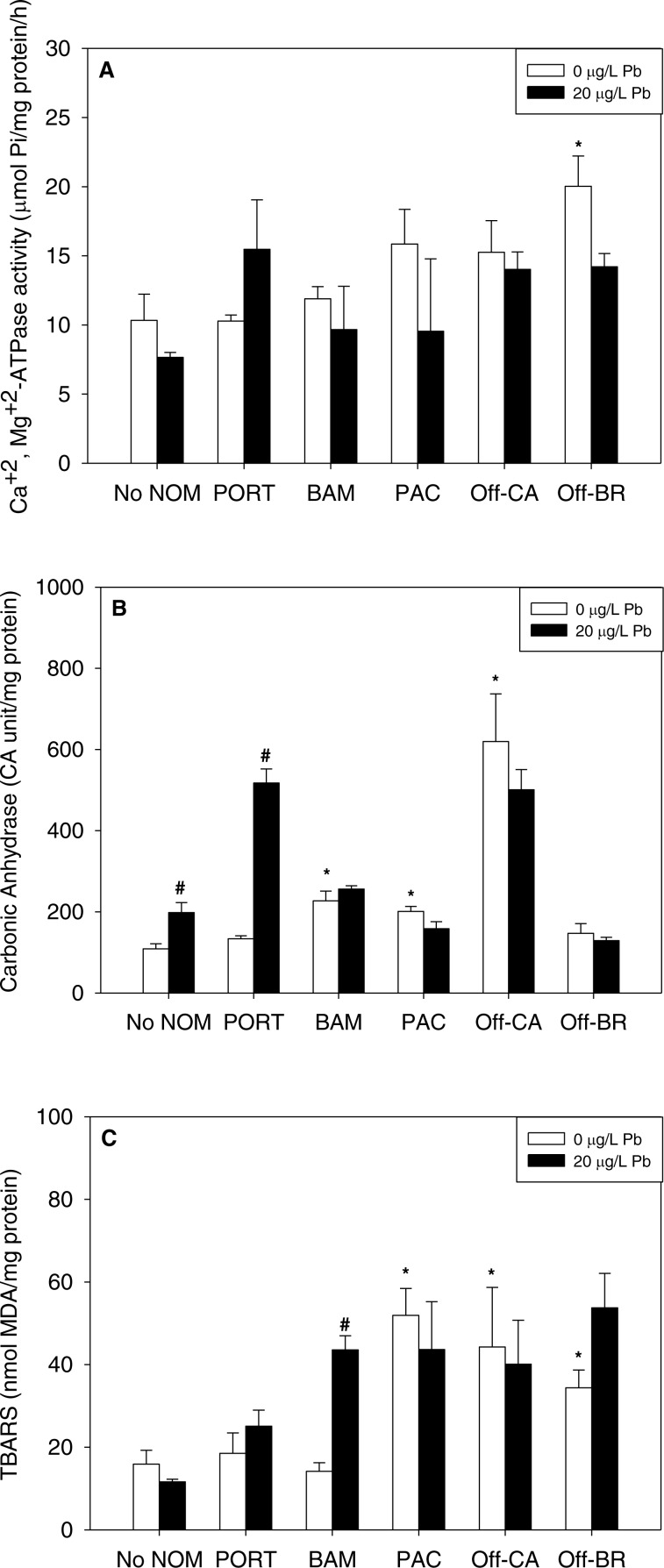
(A) Ca^2+^+Mg ^2+^-ATPase activity, (B) carbonic anhydrase activity, and (C) lipid peroxidation, quantified as TBARS, of *M. galloprovincialis* larvae exposed to lead (20 µg/L) for 48 h at te beginning of development. The control condition (Bamfield sea water with no added NOM or metal) and isolated NOMs exposures alone (no added metal) are represented by open bars. Treatments in which lead was added are represented by black bars. Mean values which are significantly different from their respective NOM alone controls are marked with #; mean values for isolated NOM exposures alone which are significantly different from the absolute control condition are represented by an asterisk. PORT, Port; BAM, Bamfield; PAC, Pachena; Off-CA, Offshore Canada; Off-BR, Offshore Brazil. Data are means ± standard error (*N* = 3 replicates of 2,500 larvae each).

**Figure 4:**
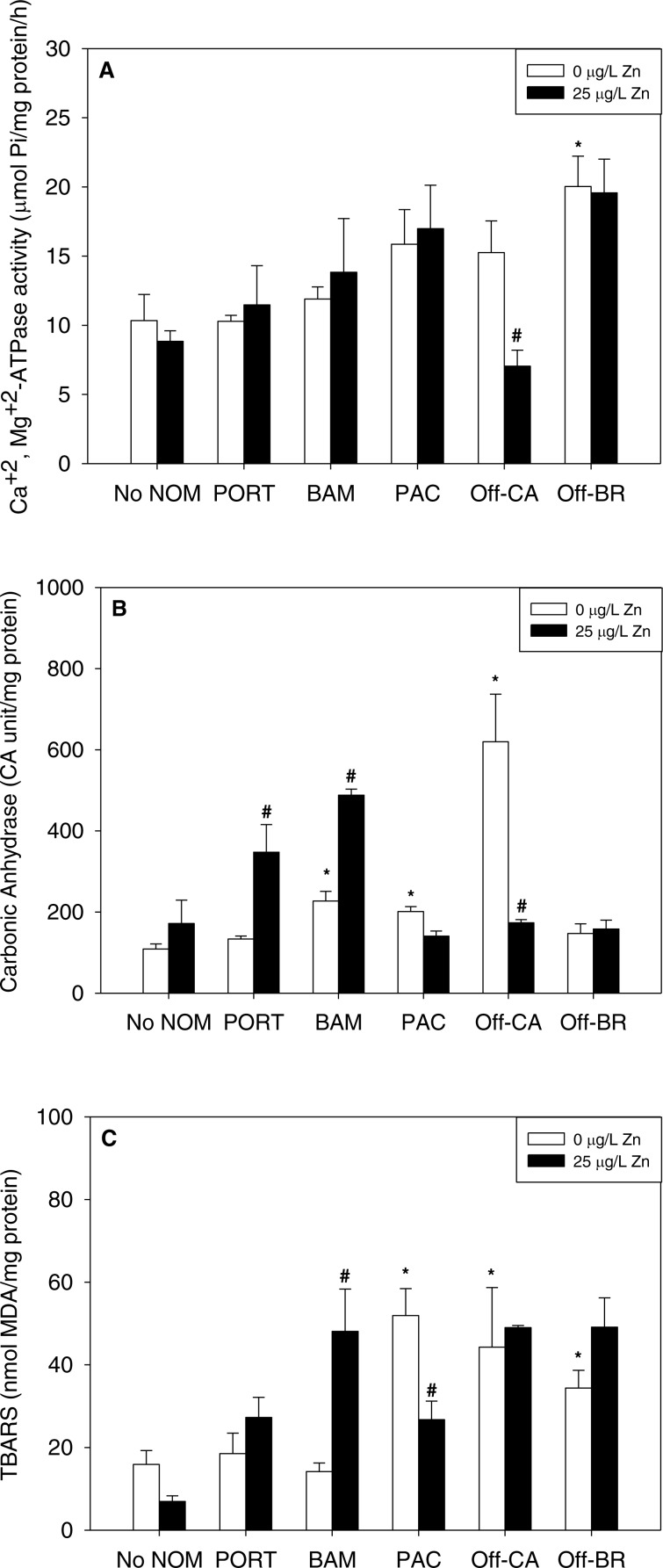
(A) Ca^2+^+Mg^2+^-ATPase activity, (B) carbonic anhydrase activity, and (C) lipid peroxidation, quantified as TBARS, of *M. galloprovincialis* larvae exposed to zinc (25 µg/L) for 48 h at the beginning of development. The control condition (Bamfield sea water with no added NOM or metal) and isolated NOMs exposures alone (no added metal) are represented by open bars. Treatments in which lead was added are represented by black bars. Mean values which are significantly different from their respective NOM alone controls are marked with #; mean values for isolated NOM exposures alone which are significantly different from the absolute control condition are represented by an asterisk. PORT, Port; BAM, Bamfield; PAC, Pachena; Off-CA, Offshore Canada; Off-BR, Offshore BR. Data are means ± standard error (*N* = 3 replicates of 2,500 larvae each).

Notably, there were no inhibitory effects of metals on enzyme activities or increases in lipid peroxidation when tested in sea water lacking added NOM. Exposure to copper, lead, or zinc did not alter Ca^2+^ + Mg^2+^-ATPase activity (Figs. 2A, 3A and 4A, respectively) or lipid peroxidation (Figs. 2C, 3C and 4C, respectively) in the early life stages of *M. galloprovincialis*. However, carbonic anhydrase activities increased significantly after exposure to copper and lead (Figs. 2B and 3B), but not to zinc (Fig. 4B). A summary of comparisons between No NOM and metals has been appended in the (Table S3A) and their respective *p*-values (Table S3B).

### Combined effects of NOMs and metals

When metals were tested in combination with added NOM, the most common pattern was that the metal exerted no additional effects (33 out of 45 metal–NOM-endpoint combinations in combined metal-NOM challenges) in comparison to the effects, or lack of effects, of the NOM alone. With respect to significant effects of metals in the combined exposures (# in Figs. 2–4), in four of the combined exposure endpoints, the effect of the metal was less than that of the NOM alone, whereas, in eight cases, the metal caused additional stimulation. Thus, generally, the influence of the NOM predominated.

The two “mixed” NOMs (Port and Bamfield) showed the greatest number of enzymatic alterations after metal addition. Carbonic anhydrase activity was increased significantly by the addition of copper, lead and zinc in the presence of Port and Bamfield, with the single exception of lead with Bamfield (Figs. 2B, 3B and 4B). All three metals also increased lipid peroxidation in the presence of Bamfield NOM (Figs. 2C, 3C and 4C). Ca^2+^+Mg^2+^-ATPase activities remained similar to their respective NOM controls, except in the case of copper which decreased Ca^2+^+Mg^2+^-ATPase activity in the presence of Bamfield NOM (Fig. 2B).

Addition of metals in the presence of autochthonous NOMs (Offshore-CA and Offshore-BR) resulted in no additional effects at all with Offshore-BR but some differences with Offshore-CA. When copper was added with Offshore-CA, carbonic anhydrase activity declined, and when zinc was added with Offshore-CA, both enzymatic activities fell (Figs. 2B, 4A and 4B).

In the presence of the terrigenous NOM (Pachena), metals exerted no stimulatory effects. However, lipid peroxidation decreased with zinc exposure (Fig. 4C) but remained higher than control values (no NOM and metals addition).

## Discussion

### Overview with respect to original hypotheses

The marked effects of NOMs alone on physiological endpoints confirmed our first hypothesis that at least some of the NOMs would exert positive effects on the organisms. Indeed, significant changes were seen in seven of 15 NOM-endpoint combinations, though not all were necessarily beneficial. In this regard, we interpret stimulation of carbonic anhydrase activity and Ca^2+^+Mg^2+^ ATPase activity as positive effects (four of the seven changes) and stimulation of lipid peroxidation as a negative effect (three of the seven changes). Another unexpected result was that our second hypothesis that metals alone would exert negative effects which would differ according to the metal, was largely not supported. Indeed metals alone had no influence on Ca^2+^+Mg^2+^ ATPase activity or lipid peroxidation, whereas both copper and lead stimulated carbonic anhydrase activity. Therefore in the absence of negative effects of metals alone, our third hypothesis, that some of the NOMs would be protective, ameliorating the negative effects of the metals, became problematical. However, it is noteworthy that the most common pattern was that the metal exerted no additional effect (33 out of 45 metal–NOM-endpoint combinations in combined metal-NOM challenges) in comparison to the effect, or lack of effect, of the NOM alone. In four of the combined exposure endpoints, the effect of the added metal was less than that of the NOM alone, whereas, in eight cases, the added metal caused additional stimulation. Clearly direct effects of the NOMs were predominant in most cases. Our final hypothesis, that effects would vary both amongst the different NOMs and among the different metals, was strongly supported for the NOMs, but less strongly for the different metals, as discussed subsequently. Finally, it is important to emphasize that the current conclusions relate only to developmental status at the end of the first 48 h of mussel life (development to the D-larva stage), and that different effects of NOMs and metals may well occur in the intervening developmental events prior to 48 h, and after 48 h as veliger development proceeds subsequently.

### Characterization of NOMs and their direct effects on mussel larvae

Extraction of NOMs from five different regions resulted in three chemically distinct groups. Offshore-CA and Offshore-BR NOMs had very similar chemical characteristics typical of autochthonous origin, with large tyrosine-like and tryptophan-like components. Pachena, which is close to the outflow of a brown-water river draining evergreen forest land, had a typical terrigenous signature high in humic substances. Bamfield and Port NOMs, which would be subject to both land runoff and open ocean influences, were classified as of “mixed” terrigenous and autochthonous. PARAFAC demonstrated Pachena to be the most terrigenous with >80% humic substance contributions to total fluorescence and FI identified the off shore samples as most authochthonous (FI greater than 1.5) with Bamfield and Pachena having intermediate properties of the end-member sources. DePalma et al. (2011b) show similar ranges in optical characteristics for 71 different marine samples taken from around the coast of North America. In her study the most authochthonous NOMs had FI values in the range 1.5–2.0 and mixed sources exhibited FI values less than 1.5, and the most terrigenous sources had PARAFAC resolved humic substances greater than 80%. This chemical differentiation allowed us to expose the mussel early life stages to marine NOMs of differing quality, in order to evaluate their potential direct effects, as well as their ability to protect against metal toxicity.

The uptake of dissolved organic matter by marine organisms has been known since the classic work of Pütter (1909) although its quantitative importance has been controversial over the years (reviewed by Jorgensen, 1976). Certainly, larvae of bivalves are able to uptake glycine and other amino acids by removing them from dissolved organic matter (Manahan & Crisp, 1983). It would seem that uptake of amino acids is an important source of nutrition to larval and early settlement stages, so some of the direct effects of NOMs seen may be of nutritive origin.

The NOM exposures, by themselves, clearly demonstrated pronounced physiological effects which varied amongst the different NOMs. However, there was no clear pattern with respect to physico-chemical properties or origins of the various NOMS. Both autochthonous, optically light NOMs (Offshore-CA and Offshore-BR) and terrigenous, optically dark NOM (Pachena) resulted in significant oxidative stress as indicated by increased lipid peroxidation, whereas the intermediate NOMs (Port and Bamfield) did not affect this endpoint. Previously, oxidative stress has also been reported in freshwater amphipods exposed to NOMs from different sources, as evidenced by decreased peroxidase and increased catalase activities (Timofeyev et al., 2004; Timofeyev et al., 2006; Timofeyev & Steinberg, 2006). The first study to postulate that NOMs may have the potential to penetrate biomembranes was Münster (1985), and more recently, this was confirmed by the finding of NOMs inside the cell and even in the nucleus (Wang, Bray & Jones, 1999). Thus, either the internal presence or the subsequent metabolism of absorbed NOMs may be associated with the production of reactive oxygen species (ROS) internally, generating oxidative stress in the aquatic animals (Steinberg et al., 2003; Timofeyev et al., 2004). Alternatively, the NOMs may catalyze the production of ROS externally (e.g., Scully et al., 1996) which could be subsequently absorbed into the organism, causing oxidative damage (Abele et al., 1998; Da Rosa, Bianchini & Monserrat, 2008).

It is noteworthy that in some cases, NOMs that caused lipid peroxidation in the early life of *M. galloprovincialis* also increased Ca^2+^+Mg^2+^-ATPase (Offshore-BR) or carbonic anhydrase activities (Offshore-CA and Pachena). These enzymatic alterations could be related to oxidative stress stimulating tyrosine phosphorylation which has been described as a “key-process” in the transduction and regulation of enzyme activity in diverse cell types (Nakashima et al., 2002). However increased carbonic anhydrase activity was also caused by Bamfield NOM (but not by Port) where lipid peroxidation was not seen. Alternatively, the increases in enzyme activity may reflect direct effects on ion transport processes due to interaction of these amphiphilic substances to external organism surfaces, thereby modifying membrane characteristics, such as surface charge (Campbell, Twiss & Wilkinson, 1997), transepithelial potential (McGeer et al., 2002; Galvez et al., 2008) and membrane permeability (Vigneault et al., 2000; Sanchez-Marin, Bellas & Mubiana, 2011).

### Direct effects of metals alone on mussel larvae

Metals interact with specific binding sites on the external epithelia of freshwater or seawater animals and compete with essential ions, such as sodium and calcium, to enter into the organism (Niyogi & Wood, 2004; Nogueira et al., 2013; Dudev & Lim, 2014). The anticipated negative effects of metals alone (inhibition of carbonic anhydrase and/or Ca^2+^+Mg^2+^ ATPase activities, increased lipid peroxidation) were not seen; indeed both copper and lead stimulated carbonic anhydrase activity. There are several possible explanations. Firstly, these effects have been described previously mainly in freshwater organisms (see Introduction), and the very different physiologies of seawater organisms, as well as the very different physical chemistry of sea water, may prevent their occurrence. Secondly, there is considerable evidence that the effects caused by metals are both time-dependent and concentration-dependent (e.g., McDonald & Wood, 1993; Jorge et al., 2013; Lionetto et al., 2006; Lionetto et al., 2012). For example, in early development tests with another very sensitive marine organism, the purple sea urchin, all three of the metals tested here clearly interfered with calcium metabolism, but at different times of exposure endpoints such as calcium uptake rate, whole body calcium concentration, and Ca^2+^-ATPase activity were variously not affected, inhibited, or stimulated (Tellis et al., 2013; Tellis et al., 2014). The latter may be interpreted as a compensatory response to earlier enzyme inhibition or developmental delay, and this may also be the explanation for the increased carbonic anhydrase activity caused by copper and lead.

### Combined effects of NOMs and metals

Given the lack of negative effects caused by metals alone, it becomes problematical to assess whether NOMs protected against metal toxicity. The addition of metals in the presence of “mixed” NOMs (Port and Bamfield) caused additional stimulation of carbonic anhydrase activity (except for Bamfield and lead) and lipid peroxidation (only Bamfield NOM with all three metals). This could be interpreted as either a lack of protection, or an exacerbation of toxic effect, reflecting the intermediate or mixed nature of the chemical structures.

The comparison among NOMs used in this study shows Pachena as the NOM with the highest amount of humic substances and darkest color (Fig. S1) which indicates a higher amount of phenolic rings and a greater ability to chelate metals compared to organic matter of lighter color (Gheorghiu et al., 2010; Wood, Al-Reasi & Smith, 2011; Baken et al., 2011; Al-Reasi, Wood & Smith, 2011; Al-Reasi, Smith & Wood, 2012). However, with respect to Pachena NOM, the only significant metal effect observed was a decrease in lipid peroxidation in the zinc treatment. Other significant metal effects were seen with Offshore-CA, where reduced stimulations of Ca^2+^ + Mg^2+^-ATPase and carbonic anhydrase activities were seen when Zn was added to Offshore-CA. Offshore-CA NOM had a lesser amount of humic substance-like components but more of the proteinaceous materials (tryptophan-like and tyrosine-like). As shown in some studies, these types of compounds affect directly the capacity to bind metals (Schwartz, Curtis & Playle, 2004; DePalma et al., 2011a; DePalma et al., 2011b).

Thus, as in fresh water (see Introduction), our study reinforces the importance of the quality of NOMs both in their direct effects and their interactions with metal effects in a model marine organisms. All marine NOMs are not alike. The latter has previously been shown in several other seawater studies (Nadella et al., 2009; Nadella et al., 2013; Blewett et al., 2016). However, the “rules” which have become clear for predicting potency from optical characteristics in fresh water (e.g., Galvez et al., 2008; Baken et al., 2011; Al-Reasi, Wood & Smith, 2011; Wood, Al-Reasi & Smith, 2011), do not seem to apply in sea water. Clearly further work in this area is needed.

### Regulatory significance

Currently, computational models such as the Biotic Ligand Model (Di Toro et al., 2001; Santore et al., 2001; Niyogi & Wood, 2004) that predict the site-specific toxicity of metals or derive site-specific ambient water quality criteria in fresh water treat all NOMs as the same. They represent organic matter as dissolved organic carbon (DOC) and relate the protective abilities of NOM simply to the DOC concentration, regardless of the chemical composition of the NOM (Di Toro et al., 2001; Santore et al., 2001; Niyogi & Wood, 2004). Nevertheless, there is emerging evidence (discussed in the Introduction) that differences in NOM chemistry should be taken into account in fresh water. In sea water (again as discussed in the Introduction), the situation is less clear, although several recent studies with copper have concluded that that it is not necessary to take NOM chemistry into account (Arnold, Santore & Cotsifas, 2005; Arnold, Cotsifas & Corneillie, 2006; DePalma et al., 2011a; DePalma et al., 2011b; Tait et al., 2016).

**Figure 5:**
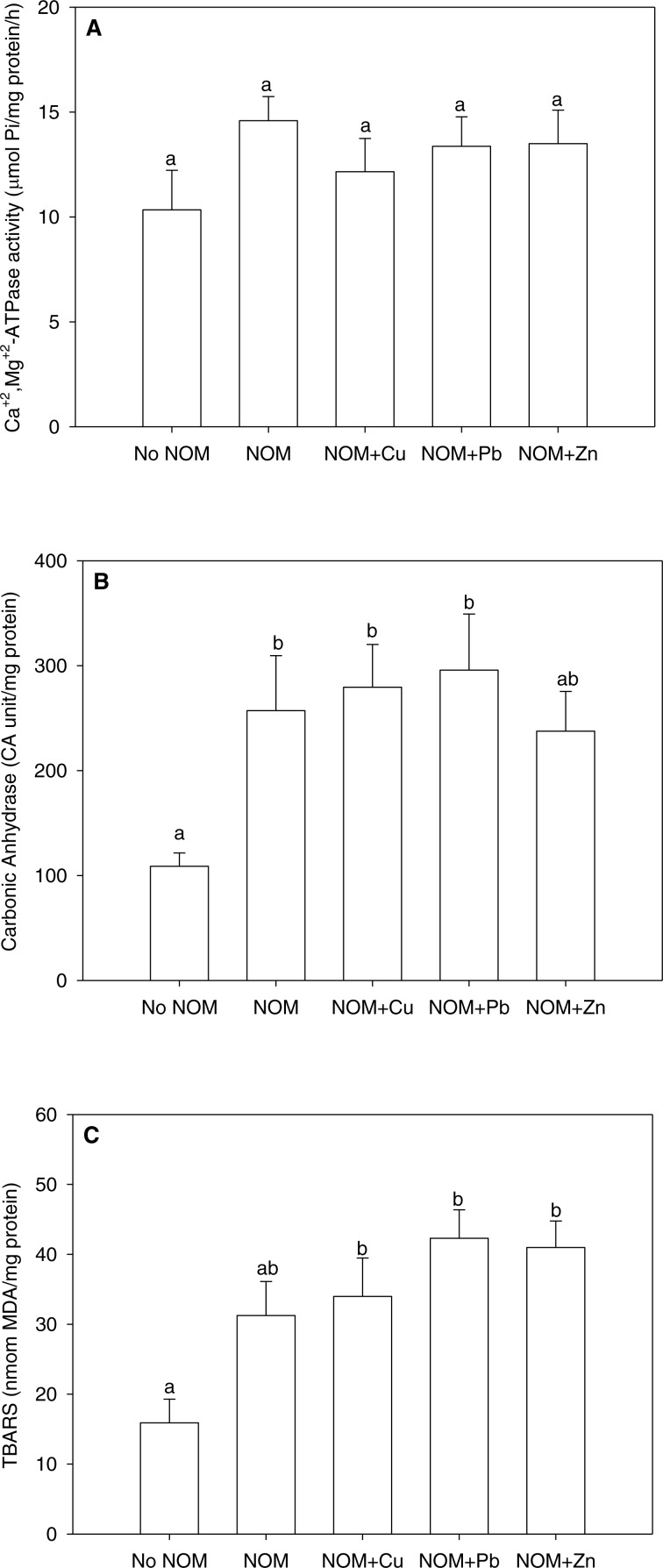
The results of an analysis in which all NOMs were considered the same and pooled. (A) Ca^2+^+Mg^2+^-ATPase activity, (B) carbonic anhydrase activity, and (C) lipid peroxidation of *M. galloprovincialis* larvae exposed to copper (6 µg/L), lead (20 µg/L), or zinc (25 µg/L) for 48 h at the beginning of development. See the ‘Discussion’ section for details. Mean values represent control condition (Bamfield sea water with no added NOM or metal), average of all NOM exposures (no added metal), and NOMs with additional copper, lead or zinc. No NOM, control condition; NOM, natural organic matter only added; NOM + Cu, natural organic matter plus copper; NOM + Pb, natural organic matter plus lead; NOM + Zn, natural organic matter plus zinc. Bars sharing the same letter are not significantly different. Data are means ± standard error (*N* = 15 replicates of 2,500 larvae each).

With this background in mind , we re-analysed the current data set considering all NOMs as a single source, thereby pooling all NOM data together (Figs. 5A –5C). The pooled data could not be normalized by standard transformations, so a one-way non-parametric ANOVA was used, followed by multiple comparisons test. As comparison to Figs. 2–4 will indicate, rather different conclusions would be reached. For Ca^2+^+Mg^2+^-ATPase activity, the combined NOM treatment, alone or in combination with various metals, had no significant effect (Fig. 5A). However for carbonic anhydrase activity (Fig. 5B) and lipid peroxidation (Fig. 5C), the combined NOM treatments resulted in substantial (two-fold) increases relative to the control with no added NOM, and none of the three metals exerted any additional effects—i.e., the influence of NOM alone completely dominated, and the presence and/or nature of the metal did not matter. Clearly, many of the subtle physiological differences seen with individual NOMs were lost. Whether this will matter with respect to the toxicological effects of these metals, as assessed by classic mortality tests, remains to be seen. In terms of enviromental significance, these results suggest that more emphasis should be placed on the site-specific nature and typical concentration of NOM in selecting sites for mussel aquaculture, as well as in interpreting whether metal levels in inshore waters are a threat to these model organisms, which play important roles in setting marine water quality standards.

##  Supplemental Information

10.7717/peerj.3141/supp-1 Figure S1 Supplementary information for Results: Each tube represents the final extract of the NOMs from each source. As can be seen, they can be differentiated by their distinct colours The dissolved organic carbon (DOC) concentrations of the NOMs from different sources are Pachena = 7.4 mg DOC/L, Bamfield = 6.3 mg DOC/L, Port = 7.9 mg DOC/L, Off-CA = 6.0 mg DOC/L, Off-BR= 9.1 mg DOC/L.

10.7717/peerj.3141/supp-2 Table S1 Supplementary information for Material and Methods: Schematic representation of the experimental design using nominal metal concentrations and DOC Treatments were performed in triplicate ( *N* = 3) using approximately 2500 embryos each.

10.7717/peerj.3141/supp-3 Table S2 Supplementary information for Material and Methods: *P*-values from comparisons between the absolute control condition (Bamfield sea water with no added NOM) and NOMs after 48 h exposure. Bold type indicates statistical differences

10.7717/peerj.3141/supp-4 Table S3 Supplementary information for Results: (A) Represents Ca^2+^, Mg^2+^-ATPase activity (µmol Pi/mg protein/h), carbonic anhydrase activity (CA unit/mg protein/h), and lipid peroxidation, quantified as TBARS (nmol MDA/mg protein), of *M. galloprovincialis* larvae exposed to copper (6 µg/L), lead (20 µg/L) and zinc (25 µg/L) for 48 h at the beginning of development Asterisks (*) indicate only significant differences between metals and the absolute control condition (Bamfield sea water with no added NOM). (B) Represent p-values from comparisons between the absolute control condition (Bamfield sea water with no added NOM). Bold type indicates statistical differences. The significance level adopted was 95% ( *α* = 0.05).

## Additional Information and Declarations

### Data Availability

The following information was supplied regarding data availability: The raw data has been supplied as a  Supplementary File.
