# Supplementary figures and images for: Physiological effects of five different marine natural organic matters (NOMs) and three different metals (Cu, Pb, Zn) on early life stages of the blue mussel (Mytilus galloprovincialis)
Source: PeerJ. 2017 Apr 12;5:e3141. doi: 10.7717/peerj.3141 (PMC5391792; doi:10.7717/peerj.3141)

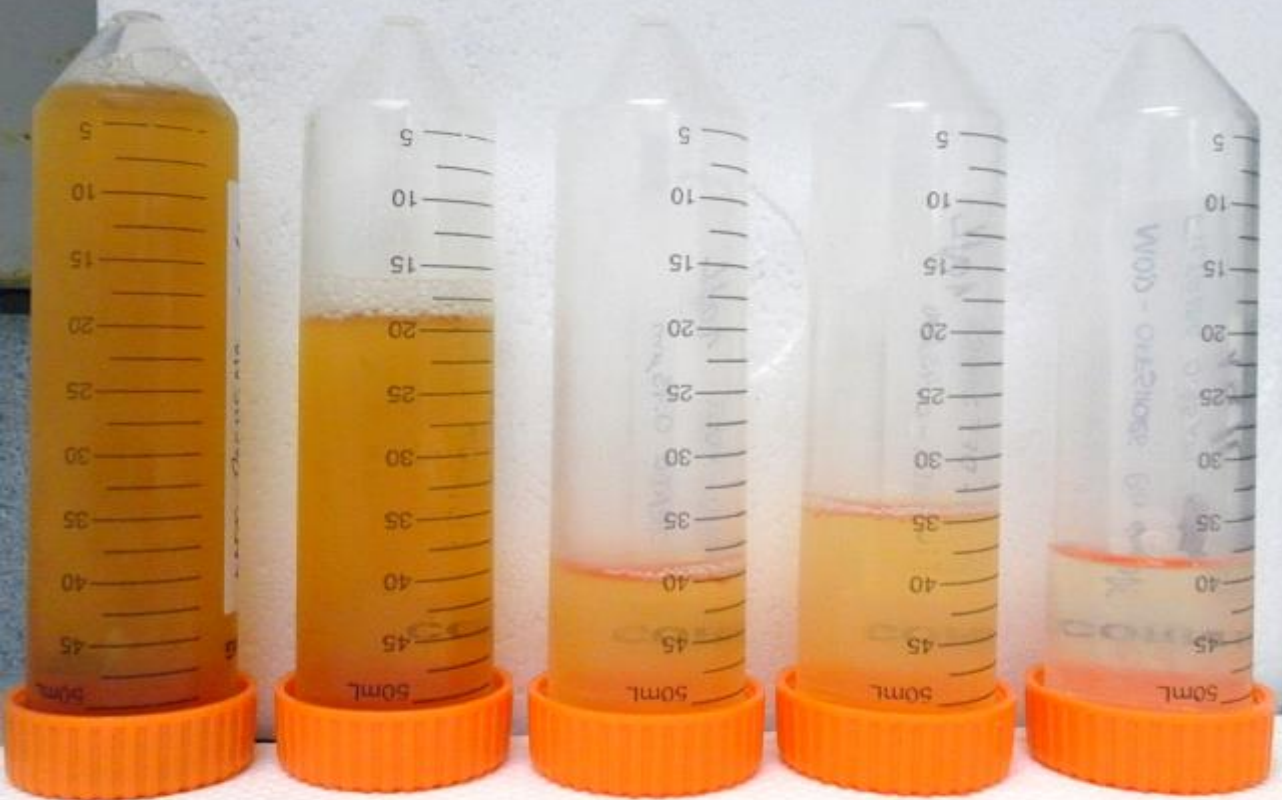

**Pachena**

**Bamfield**

**Port**

**Offshore-CA**

**Offshore-BR**

Supplement: Figure S1 — The dissolved organic carbon (DOC) concentrations of the NOMs from different sources are Pachena = 7.4 mg DOC/L, Bamfield = 6.3 mg DOC/L, Port = 7.9 mg DOC/L, Off-CA = 6.0 mg DOC/L, Off-BR= 9.1 mg DOC/L. [file peerj-05-3141-s001.pdf]
