# Supplementary material for: Physiological effects of five different marine natural organic matters (NOMs) and three different metals (Cu, Pb, Zn) on early life stages of the blue mussel (Mytilus galloprovincialis)
Source: PeerJ. 2017 Apr 12;5:e3141. doi: 10.7717/peerj.3141 (PMC5391792; doi:10.7717/peerj.3141)
Supplement: Table S1 — Treatments were performed in triplicate ( N = 3) using approximately 2500 embryos each. [file peerj-05-3141-s002.docx]

| Treatments |
| --- |
| *Control (Bamfield water)* |
| *6 µg/L Copper* |
| *20 µg/L Lead* |
| *25 µg/L Zinc* |
| *6 µg/L Cu + 8 mg DOC/L PORT* |
| *6 µg/L Cu + 8 mg DOC/L BAM* |
| *6 µg/L Cu + 8 mg DOC/L PAC* |
| *6 µg/L Cu + 8 mg DOC/L Off-CA* |
| *6 µg/L Cu + 8 mg DOC/L Off-BR* |
| *20 µg/L Pb + 8 mg DOC/L PORT* |
| *20 µg/L Pb + 8 mg DOC/L BAM* |
| *20 µg/L Pb + 8 mg DOC/L PAC* |
| *20 µg/L Pb + 8 mg DOC/L Off-CA* |
| *20 µg/L Pb + 8 mg DOC/L Off-BR* |
| *25 µg/L Zn + 8 mg DOC/L PORT* |
| *25 µg/L Zn + 8 mg DOC/L BAM* |
| *25 µg/L Zn + 8 mg DOC/L PAC* |
| *25 µg/L Zn + 8 mg DOC/L Off-CA* |
| *25 µg/L Zn + 8 mg DOC/L Off-BR* |
