# Supplementary material for: Physiological effects of five different marine natural organic matters (NOMs) and three different metals (Cu, Pb, Zn) on early life stages of the blue mussel (Mytilus galloprovincialis)
Source: PeerJ. 2017 Apr 12;5:e3141. doi: 10.7717/peerj.3141 (PMC5391792; doi:10.7717/peerj.3141)
Supplement: Table S2 [file peerj-05-3141-s003.docx]

|  | *Port* | *Bamfield* | *Pachena* | *Offshore-CA* | *Offshore-BR* |
| --- | --- | --- | --- | --- | --- |
| *Ca^2+^,Mg^2+^-ATPase* | 1 | 0.958 | 0.184 | 0.265 | 0.012 |
| *Carbonic anhydrase* | 0.77 | 0.006 | 0.017 | <0.001 | 0.497 |
| *Lipid peroxidation* | 0.992 | 0.998 | 0.017 | 0.049 | 0.028 |
