# Supplementary material for: Physiological effects of five different marine natural organic matters (NOMs) and three different metals (Cu, Pb, Zn) on early life stages of the blue mussel (Mytilus galloprovincialis)
Source: PeerJ. 2017 Apr 12;5:e3141. doi: 10.7717/peerj.3141 (PMC5391792; doi:10.7717/peerj.3141)
Supplement: Table S3 — Asterisks (*) indicate only significant differences between metals and the absolute control condition (Bamfield sea water with no added NOM). (B) Represent p-values from comparisons between the absolute control condition (Bamfield sea water with no added NOM). Bold type indicates statistical differences. The significance level adopted was 95% ( α = 0.05). [file peerj-05-3141-s004.docx]

(A)

|  | *No NOM* | *Cu* | *Pb* | *Zn* |
| --- | --- | --- | --- | --- |
| *Ca^2+^,Mg^2+^-ATPase* | 10.33 ± 1.88 | 8.71 ± 0.49 | 7.65 ± 0.35 | 8.83 ± 0.75 |
| *Carbonic anhydrase* | 108.79 ± 12.79 | 243.31 ± 42.05* | 198.32 ± 24.45* | 171.68 ± 57.54 |
| *Lipid peroxidation* | 15.90 ± 3.37 | 6.40 ± 0.33 | 11.63 ± 0.63 | - 1. ± 1.36 |

(B)

|  | *Cu* | *Pb* | *Zn* |
| --- | --- | --- | --- |
| *Ca^2+^,Mg^2+^-ATPase* | 0.372 | 0.200 | 0.446 |
| *Carbonic anhydrase* | 0.017 | 0.017 | 0.268 |
| *Lipid peroxidation* | 0.118 | 0.282 | 0.070 |
